# The roles of STP and LTP in synaptic encoding

**DOI:** 10.7717/peerj.3

**Published:** 2013-02-12

**Authors:** Arturas Volianskis, Graham L. Collingridge, Morten S. Jensen

**Affiliations:** 1 Institute of Biomedicine, University of Aarhus, Denmark; 2 Centre for Synaptic Plasticity, University of Bristol, United Kingdom

**Keywords:** Short-term potentiation, Long-term potentiation, Synaptic transmission, Hippocampal slices, CA1, Schaffer collaterals, Synaptic encoding and decoding, Learning and memory

## Abstract

Long-term potentiation (LTP), a cellular model of learning and memory, is generally regarded as a unitary phenomenon that alters the strength of synaptic transmission by increasing the postsynaptic response to the release of a quantum of neurotransmitter. LTP, at CA3-CA1 synapses in the hippocampus, contains a stimulation-labile phase of short-term potentiation (STP, or transient LTP, t-LTP) that decays into stable LTP. By studying the responses of populations of neurons to brief bursts of high-frequency afferent stimulation before and after the induction of LTP, we found that synaptic responses during bursts are potentiated equally during LTP but not during STP. We show that STP modulates the frequency response of synaptic transmission whereas LTP preserves the fidelity. Thus, STP and LTP have different functional consequences for the transfer of synaptic information.

## Introduction

The mechanisms by which synapses store information have been explored extensively and much is known, especially with regard to long-term potentiation (LTP) (Bliss & Lomo, 1973) at the CA3-CA1 synapses in the hippocampus (Bliss & Collingridge, 1993; Collingridge & Bliss, 1995; Malenka & Nicoll, 1999; Malinow & Malenka, 2002; Lauri et al., 2007; Kerchner & Nicoll, 2008). Although much of the LTP research has been conducted at these synapses in hippocampal slices, the functional consequences of such potentiation are still under debate (Stevens, 1998; Albright et al., 2000; Lisman & Raghavachari, 2006; Lauri et al., 2007).

Application of brief, high frequency trains of electrical stimuli to the Schaffer collaterals evokes three phases of potentiation at the CA3-CA1 synapses. Post-tetanic potentiation (PTP), mediated by an increase in the probability of neurotransmitter release (*P*_*R*_), is the first in the cascade (Zucker, 1999; Zucker & Regehr, 2002). PTP is caused by a presynaptic accumulation of [Ca^2+^]_*i*_ and decays rapidly upon its clearance. PTP, in the CA1 area, is independent of the N-methyl-D-aspartate (NMDA) receptor activation during the tetanus and can be studied in isolation from the NMDA receptor dependent types of potentiation (Collingridge, Kehl & McLennan, 1983; Anwyl, Lee & Rowan, 1988; Malenka et al., 1988; Anwyl, Mulkeen & Rowan, 1989; Malenka, Lancaster & Zucker, 1992; Stevens, Tonegawa & Wang, 1994; Tsien, Huerta & Tonegawa, 1996; Volianskis & Jensen, 2003). When induced in isolation PTP decays passively and disappears within a time period of about two minutes (Stevens, Tonegawa & Wang, 1994; Volianskis & Jensen, 2003). In addition to the PTP, high frequency stimulation induces two phases of NMDA receptor dependent LTP (Collingridge, Kehl & McLennan, 1983; Anwyl, Lee & Rowan, 1988; Malenka et al., 1988; Anwyl, Mulkeen & Rowan, 1989; Malenka, Lancaster & Zucker, 1992; Stevens, Tonegawa & Wang, 1994; Tsien, Huerta & Tonegawa, 1996; Volianskis & Jensen, 2003). Short-term potentiation (STP, or transient LTP, t-LTP) is an unstable phase, which declines over a period of about half an hour and leads to a sustained level of up-regulated neurotransmission, which is most commonly referred to as LTP (though sometimes as early-LTP, e-LTP). STP and LTP are generally regarded as a unitary phenomenon (Gustafsson et al., 1989; Gustafsson & Wigstrom, 1990; Hanse & Gustafsson, 1994) that alters the efficiency of synaptic transmission by increasing the postsynaptic response to the release of a quantum of neurotransmitter.

STP and LTP are usually investigated in terms of the synaptic response to single stimuli delivered at low frequency (0.033–0.1 Hz, Bliss & Lomo, 1973; Andersen et al., 1977; Bliss & Collingridge, 1993; Collingridge & Bliss, 1995; Malenka & Nicoll, 1999; Malinow & Malenka, 2002; Lauri et al., 2007; Kerchner & Nicoll, 2008). STP decreases actively in response to stimulation and in this way it differs from both PTP and LTP (Volianskis & Jensen, 2003). Notably, synaptic activation at low frequency mimics poorly the natural patterns of neuronal communication in the hippocampus, as neurons typically communicate with brief high frequency bursts of action potentials (Dobrunz & Stevens, 1999). Indeed, it has been shown that synaptic responses to high frequency bursts of the presynaptic action potentials are not always equally potentiated during LTP (Markram & Tsodyks, 1996; Yasui et al., 2005), although sometimes they are (Pananceau, Chen & Gustafsson, 1998; Selig, Nicoll & Malenka, 1999). Consequently, the functional implications of the long-lasting changes in synaptic transmission cannot be predicted by analyses of the single synaptic responses alone (Markram & Tsodyks, 1996; Tsodyks & Markram, 1997; Dobrunz & Stevens, 1999).

It is currently not known whether the synaptic responses during high frequency bursts are affected similarly during STP and LTP. We report here that STP and LTP have dramatically different consequences during high frequency synaptic transmission, such that STP alters the temporal characteristics of the burst whereas LTP maintains the synaptic fidelity.

## Materials and Methods

Experiments were conducted according to the national and the EU guidelines for animal care, according to procedures certified by the University of Aarhus. Extracellular f-EPSPs were recorded in response to control stimulation (0.067 Hz), in hippocampal slices from adult Wistar rats (about 6 month old) as previously described (Volianskis & Jensen, 2003). Potentiation was induced by theta-burst stimulation (4 pulses at 100 Hz repeated 10 times at 5 Hz). f-EPSPs were analysed in terms of post-tetanic changes in the rates of rise of the potentials relative to the pre-tetanic periods of baseline responses (set to 100%). Potentiation was expressed as gain by subtracting the baseline level (100%). Control stimulation was interposed by bursts of 7 stimuli, both before and after the induction of potentiation. Burst-evoked f-EPSPs were analyzed in terms of (1) facilitation (i.e. responses 2 to 7 were normalised to the first response) and (2) potentiation (i.e. the post-tetanic responses 1 to 7 were normalised to their respective mean responses during the baseline).

Data are presented both as single experiments and as mean values of experimental groups (±S.E.M). Student’s t tests, F test, one-way analyses of variance (ANOVA), repeated measures ANOVA (rm-ANOVA) and mixed factorial designs (mfd-ANOVA) were used for the statistic (Prism 5, GraphPad). Bonferroni’s (Bmc-test) adjustments were made for multiple comparisons; significant differences were set at *P* < 0.05 for all tests.

## Results

**Figure 1:**
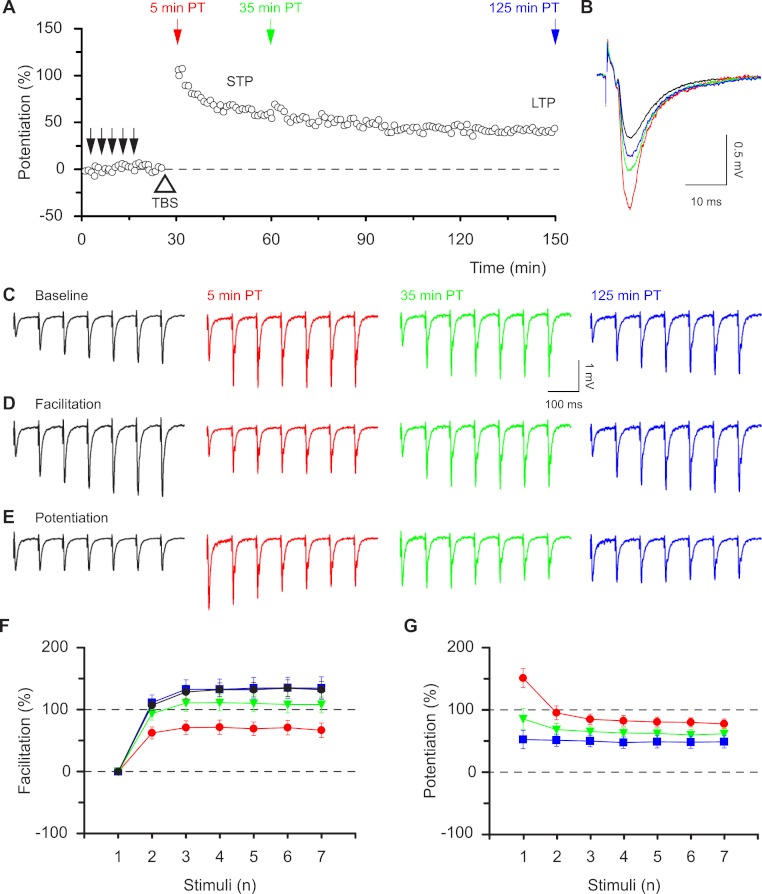
Synaptic responses within high frequency bursts are regulated by LTP in a time-dependent manner. (A) Potentiation, induced by theta-burst stimulation (TBS, open triangle), consisted of a declining phase (STP) and a sustained phase (LTP). Brief bursts (7 stimuli, 12.5 Hz) were applied at times indicated by the coloured arrows before (black) and at various times post-tetanus (PT, red, green and blue). Results, in this and the following figures, are colour-coded according to the arrows. (B) Representative f-EPSPs during low frequency transmission before and after the induction of potentiation. (C–E) Representative f-EPSPs during the bursts plotted in absolute values (C), normalised to the first response in each burst, i.e. facilitation (D) or normalised to the corresponding response in the baseline, i.e. potentiation (E). F & G show how facilitation (F) and potentiation (G) alter depending on the time after TBS (mean ± S.E.M., *n* = 12).

To evaluate the functional consequences of STP and LTP we followed the time-course of potentiation by applying low frequency stimulation (0.067 Hz, Fig. 1A) and delivered brief high frequency test bursts (seven stimuli at 12.5 Hz) at specific time points before and after induction of potentiation. Potentiation of field excitatory postsynaptic potentials (f-EPSPs) in response to single stimuli was biphasic (Figs. 1A and 1B). It consisted of a period of short-term potentiation (STP), which peaked at approximately 120% over baseline and declined in about one hour to a stable level of sustained long-term potentiation (approximately 40% over baseline, LTP). We determined the pattern of synaptic transmission during the test bursts and found a progressive increase in response amplitude during the burst in baseline conditions (Fig. 1C). STP and LTP differentially affected this pattern of activity as can be readily seen in the facilitation plots, which express the level of facilitation within the burst (each response in the train is normalised to the initial response, Fig. 1D). Facilitation was reduced at 5 min following induction but was restored to its initial level at 125 min post induction (Fig. 1D). Facilitation was quantified (seventh response divided by first response in train) during baseline (132 ± 14%) and at three time points after delivery of theta burst stimulation (5 min: 66 ± 12%, *P* < 0.001; 35 min: 108 ± 14%, *P* < 0.05; 125 min: 134 ± 18%, *P* > 0.05; rm-ANOVA, Bmc-test compared with initial baseline), which correlated in time with STP, STP + LTP and LTP, respectively (Fig. 1F).

The differential effects of STP and LTP on responses during bursts are demonstrated by potentiation plots, which express the size of each response in the post-induction burst relative to the corresponding response in the baseline burst (Fig. 1E). The analysis of the first response in a burst is equivalent to the conventional LTP analysis of single evoked responses. However, potentiation plots permit quantitative assessment of whether all responses in a burst are potentiated equally. Notably, the initial response during the STP phase showed a much lager initial potentiation and a much greater response decrement with time, compared to the subsequent responses within the bursts (Figs. 1E and 1G). The level of potentiation of the first and seventh responses expressed relative to their respective baseline responses was, respectively, 151 ± 16% and 78 ± 8% (*P* < 0.001, paired *t* test) at 5 min post induction compared with 53 ± 15% and 49 ± 10% (*P* = 0.6) at 125 min post induction. Thus the effects of STP on responses during bursts are nonlinear whereas a linear amplification is achieved by LTP. These results show that STP and LTP differentially affect the ability of synapses to respond to high frequency bursts depending on the time following induction.

**Figure 2:**
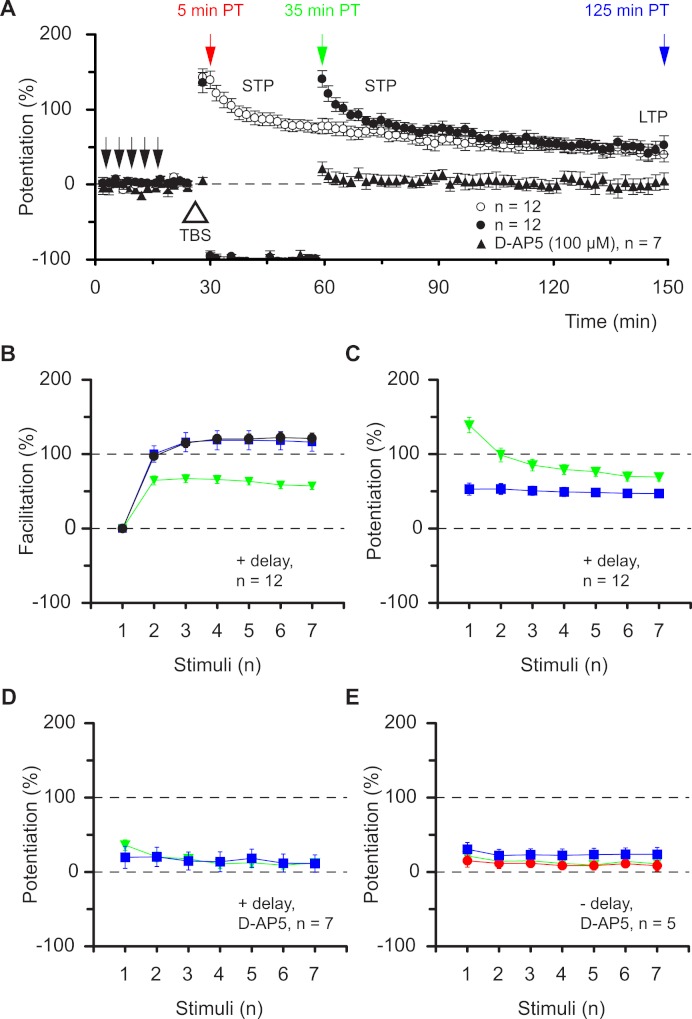
Alterations in synaptic dynamics can be stored during periods of inactivity. (A) Pooled data from experiments as in Fig. 1 superimposed upon equivalent experiments in which there was a 30 min gap in stimulation, commencing 4 min after TBS. Note how the STP phase is “stored” in time. Also superimposed are “gap” experiments in which TBS was delivered in the presence of an NMDAR antagonist. (B & C), plot pooled data for facilitation and potentiation in the experiments with gaps. (D & E) Experiments performed in D-AP5 show that induction of all plasticity depended on activation of NMDARs.

A remarkable property of STP makes the situation more complex. STP does not decline passively (Volianskis & Jensen, 2003), rather stimulation is needed to evoke its decay (filled circles vs. open circles, Fig. 2A). Notably, in experiments in which testing of STP was delayed by 30 min, the level of facilitation of the seventh response during the burst was 57 ± 5% at 35 min post induction (green triangles Fig. 2B), i.e. similar to that at 5 min post induction (66 ± 12%, red circles Fig. 1F, *P* = 0.5, *t* test) in experiments without the delay in stimulation and smaller than the temporally equivalent estimate (108 ± 14%, green triangles Fig. 1F, *P* < 0.01, *t* test). Accordingly, potentiation of the burst responses measured after a 30 min delay in stimulation was nonlinear (green triangles, Fig. 2C) and very similar to the potentiation recorded without the delay (Fig. 1G, red circles). The seven burst responses were equally potentiated during the LTP phase of both sets of experiments (blue squares, Figs. 2C and 1G). All synaptic plasticity studied in these experiments was induced through activation of N-methyl-D-aspartate receptors (NMDARs, Figs. 2A, 2D and 2E). These findings show that the pattern of the synaptic response to a high frequency input is determined not by the time after induction of potentiation but rather by the type of potentiation (STP or LTP) and by the subsequent history of synaptic transmission.

**Figure 3:**
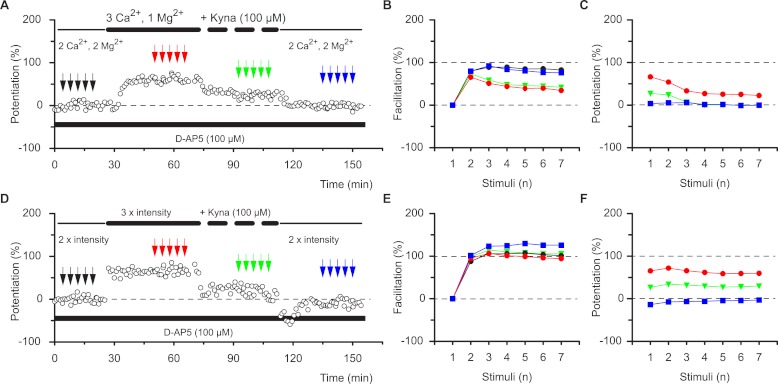
The effects of STP are mimicked by increasing *P*_*R*_. Altering the divalent cation ratio (A–C) increases synaptic transmission and mimics the effects of TBS on the burst profile during STP, whereas blockage of AMPA receptors with kynurenic acid (Kyna) reduces single shock responses but does not alter the burst profile. (B–F) Increase in the stimulation intensity enhances synaptic transmission that can be reduced by kynurenic acid and these procedures do not alter the burst profile.

So how can two phases of potentiation, with different functional characteristics, coexist at the same population of synapses? Potentiation of neurotransmission can be mediated through various synaptic mechanisms that do not have to be mutually exclusive (Bliss & Collingridge, 1993; Collingridge & Bliss, 1995; Malenka & Nicoll, 1999; Malinow & Malenka, 2002; Lauri et al., 2007; Kerchner & Nicoll, 2008). Presynaptic changes in the probability of neurotransmitter release (*P*_*R*_) affect high-frequency neurotransmission nonlinearly. In contrast, an increase in the number or single channel conductance of α-amino-3-hydroxy-5-methyl-4-isoxolepropionic acid receptors (AMPARs) can be expected to result in a linear scaling of synaptic responses during bursts of activity. There is evidence that each of these mechanisms can account for potentiation (Markram & Tsodyks, 1996; Tsodyks & Markram, 1997; Pananceau, Chen & Gustafsson, 1998; Selig, Nicoll & Malenka, 1999; Yasui et al., 2005). However, no single mechanism has been universally accepted and with few exceptions the entire LTP process has been assumed to result from a single mode of expression (Gustafsson et al., 1989; Gustafsson & Wigstrom, 1990; Hanse & Gustafsson, 1994). Our observations could be explained by the co-existence of presynaptic and postsynaptic mechanisms that account for STP and LTP, respectively. To test this hypothesis we altered *P*_*R*_ by changing the Ca^2+^/Mg^2+^ ratio in the perfusion medium. As expected, an increase in the Ca^2+^/Mg^2+^ ratio, which increases *P*_*R*_, resulted in an increase in the response during single-shock stimulation (Fig. 3A, and additionally in 11 slices). These changes were accompanied by both a decrease in facilitation (Fig. 3B, red circles vs. black circles) and by nonlinear potentiation of the responses during the bursts (red circles Fig. 3C). In other words, increasing the Ca^2+^/Mg^2+^ ratio mimicked the effects of STP on burst responses. This change was not due to the alteration in response size *per se*. Thus, when the response was increased to a similar degree by activating a larger number of synapses (N) rather than by altering *P*_*R*_, there was a very different outcome (Fig. 3D and additionally in 8 slices). The level of facilitation within the burst was not changed by this procedure (Fig. 3E) and the potentiation was linear (i.e., each response in the burst was potentiated to the same degree, Fig. 3F). A similar result was obtained when a sub-maximal concentration of kynurenic acid (100 µM) was used to reduce the number of activated AMPARs (Fig. 3D and additionally 3 slices). Singe-shock responses were decreased, facilitation during bursts was not changed and potentiation remained linear (Figs. 3D–3F). The experiments with kynurenic acid demonstrate that presynaptic and postsynaptic mechanisms can operate independently of one another. Thus, the alteration in the facilitation profile produced by increasing the Ca^2+^/Mg^2+^ ratio is unaffected by reducing the response amplitude with kynurenic acid (Figs. 3A–3C and additionally 3 slices).

**Figure 4:**
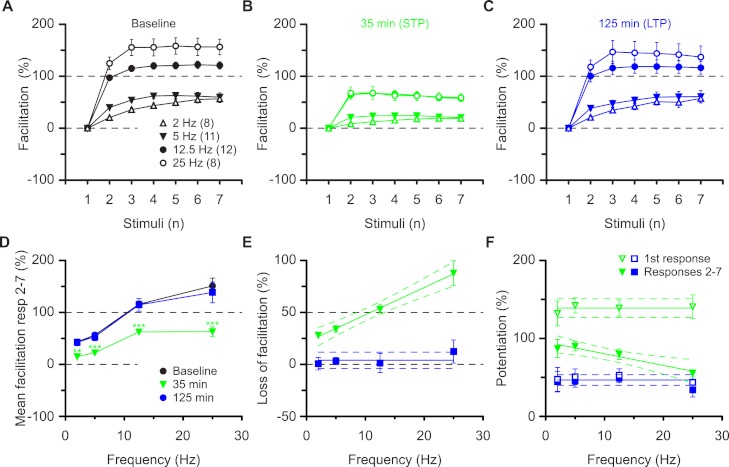
Altered burst dynamics occurs over a wide frequency range. Pooled data of facilitation plots from experiments with gaps and without application of AP5 (A–C) to illustrate how the alteration in burst dynamics during STP and LTP occurs over frequencies between 2 and 25 Hz. (D) Mean facilitation of responses 2–7 from A–C showing depression of facilitation during STP and reversal during LTP. E, shows that the reduction in facilitation during the STP burst is greatest at the highest frequency. (F) Burst responses 2-7 during STP are potentiated less at higher frequencies despite equal facilitation of the first responses. This does not happen during LTP.

These results are most compatible with the co-existence of pre- and postsynaptic forms of potentiation that respectively impart nonlinear (STP) and linear (LTP) response characteristics during high frequency synaptic transmission. These characteristics apply to the burst frequency of 12.5 Hz, which was used to ascertain the synaptic transfer function. To determine whether these characteristics can be generalised we examined a range of burst frequencies, between 2 and 25 Hz (Figs. 4A–4C). During the baseline the mean amount of facilitation of responses 2-7 depended on the burst frequency (*P* < 0.0001, ANOVA) and was more than three-fold larger at 25 Hz (151 ± 15%) than at 2 Hz (43 ± 5%, *P* < 0.001, Bmc-test, Fig. 4D). Irrespective of the burst frequency, facilitation was reduced during STP and reverted to baseline values during LTP (mfd-ANOVA, Bmc-test, Figs. 4A–4D). More importantly, dependent on the burst frequency variable amounts of facilitation were lost during STP (Fig. 4B, *P* < 0.0001, *F* test) and not during LTP (Fig. 4E, *P* = 0.4, *F* test). Thus, although the first response during STP was constant in the four experimental groups (≈140%, *P* = 0.8, *F* test, Fig. 4F) subsequent response decrement during the burst was directly related to the burst frequency (*P* < 0.01, *F* test). These findings show that STP, which is stored in synapses during periods of inactivity, can shape synaptic transfer functions during activity, based on the frequency of input. In contrast, during LTP, both the first and the subsequent burst responses are potentiated equally and irrespectively of input frequency (≈50%, *P* = 0.5, Fig. 4F).

## Discussion

The content of sensory information is encoded in the pattern of neuronal signals in terms of the frequency and the number of action potentials (Adrian, 1928). The data of the current study extend these principles to the central processing of cognitive information by demonstrating that NMDAR dependent potentiation confers synaptic connections with an ability to shape neurotransmission according to the pattern of the incoming signals. More specifically, STP amplifies synaptic signals in a non-linear, frequency-dependent manner whereas LTP evokes a linear change in synaptic gain that is independent of frequency (Pananceau, Chen & Gustafsson, 1998; Selig, Nicoll & Malenka, 1999). Thus, the two types of potentiation regulate neurotransmission differentially in that STP modulates response dynamics whereas LTP simply regulates the amplitude of synaptic events. The features of STP described here differ from other types of presynaptic plasticity (Markram & Tsodyks, 1996; Tsodyks & Markram, 1997) in that its effects have the capacity to last a long time but the process is highly labile in response to the synaptic activity that is used to probe it. Indeed, this synaptic version of Heisenberg’s uncertainty principle has been previously termed transient LTP (Volianskis & Jensen, 2003), in contrast to the sustained LTP (s-LTP), which is much more predictable (Pananceau, Chen & Gustafsson, 1998; Selig, Nicoll & Malenka, 1999).

So why hasn’t the functional difference between STP and LTP been observed before? Many studies, aimed at addressing the molecular basis of LTP, use a pairing protocol to induce potentiation and a relatively high baseline frequency to monitor the plasticity; stimulus parameters that are not conducive to evoking and observing STP. When high frequency stimuli have been used to evoke LTP and low frequency stimuli used to monitor the potentiation then a pronounced STP is often seen. In fact, STP can be induced independently from LTP both *in vitro* (Collingridge, Kehl & McLennan, 1983; Kauer, Malenka & Nicoll, 1988; Anwyl, Mulkeen & Rowan, 1989; Malenka, 1991; Colino, Huang & Malenka, 1992; Kullmann et al., 1992; Erickson, Maramara & Lisman, 2010; Volianskis et al., 2013) and *in vivo* (Buschler, Goh & Manahan-Vaughan, 2012). STP is also frequently observed when induction of LTP is unsuccessful or is blocked by manipulation of the second messenger systems, although it is not currently known whether or not all forms of STP, which are described in the literature, reflect the same underlying phenomenon (for discussion see Volianskis & Jensen, 2003).

STP is often regarded as a non-stabilised form of LTP (Gustafsson et al., 1989; Gustafsson & Wigstrom, 1990; Hanse & Gustafsson, 1994) although it has been suggested that STP and LTP are different phenomena (Kauer, Malenka & Nicoll, 1988; Malenka et al., 1988; Volianskis & Jensen, 2003; Lauri et al., 2007). In support of the latter, STP and LTP can be either co-expressed or expressed independently of each other when studied in single synapses (Debanne, Gahwiler & Thompson, 1999). A phenomenon that resembles STP has been observed after exploratory learning in rats (Moser, Moser & Andersen, 1993; Moser, Moser & Andersen, 1994) and the burst stimuli that we have utilised to induce (Larson & Lynch, 1986; Larson, Wong & Lynch, 1986) and monitor potentiation mimic the natural firing patterns of the afferent neurons in the hippocampus. The high frequency components of the induction paradigm applied in conjunction with low frequency test stimulation are optimal for STP and when a gap in stimulation is introduced the persistence of this phenomenon (i.e., t-LTP) becomes readily apparent.

The co-existence of STP and LTP, with their strikingly different functional characteristics and differing cellular mechanisms, means that synaptic plasticity at CA3-CA1 synapses is considerably more complex that hitherto envisaged. The implication of this surprising finding for cognitive processing is an important challenge for the future.
